# Improving the cytotoxicity of immunotoxins by reducing the affinity of the antibody in acidic pH

**DOI:** 10.1186/s12967-023-04210-7

**Published:** 2023-08-25

**Authors:** Xiaoyu Liu, Qingqing Tan, Jiaqi Wen, Xufei Wang, Gang Yang, Yuxiao Li, Ming Lu, Wei Ye, Anfeng Si, Sujuan Ma, Tong Ding, Luan Sun, Fang Liu, Mei Zhang, Tao Jiang, Wei Gao

**Affiliations:** 1 School of Basic Medical Sciences and Jiangsu Key Lab of Cancer Biomarkers, Prevention and Treatment, Collaborative Innovation Center for Personalized Cancer Medicine, Nanjing Medical University, 101 Longmian Road, Xuehai Building, Nanjing, 211166 Jiangsu People’s Republic of China; 2 Department of Gynecology Oncology, Changzhou Maternal and Child Health Care Hospital, Changzhou Medical Center, Nanjing Medical University, Changzhou, China; 3 Department of Endocrinology, The First Affiliated Hospital With Nanjing Medical University, Nanjing, China; 4 Department of Surgical Oncology, Jinling Hospital, Medical School of Nanjing University, 34 Yanggongjing Road, Nanjing, 210000 Jiangsu People’s Republic of China; 5 The Affiliated Changzhou Second People’s Hospital of Nanjing Medical University, Changzhou Second People’s Hospital, Changzhou Medical Center, Nanjing Medical University, Changzhou, China

**Keywords:** Immunotoxin, Low pH-responsive antigen binding, Lysosomal degradation, Toxin release, Glypican-3

## Abstract

**Background:**

Immunotoxins are antibody-toxin conjugates that bind to surface antigens and exert effective cytotoxic activity after internalization into tumor cells. Immunotoxins exhibit effective cytotoxicity and have been approved by the FDA to treat multiple hematological malignancies, such as hairy cell leukemia and cutaneous T-cell lymphoma. However, most of the internalized immunotoxin is degraded in lysosomes, and only approximately 5% of free toxin escapes into the cytosol to exert cytotoxicity. Many studies have improved immunotoxins by engineering the toxin fragment to reduce immunogenicity or increase stability, but how the antibody fragment contributes to the activity of immunotoxins has not been well demonstrated.

**Methods:**

In the current study, we used 32A9 and 42A1, two anti-GPC3 antibodies with similar antigen-binding capabilities and internalization rates, to construct scFv-mPE24 immunotoxins and evaluated their in vitro and in vivo antitumor activities. Next, the antigen-binding capacity, trafficking, intracellular protein stability and release of free toxin of 32A9 scFv-mPE24 and 42A1 scFv-mPE24 were compared to elucidate their different antitumor activities. Furthermore, we used a lysosome inhibitor to evaluate the degradation behavior of 32A9 scFv-mPE24 and 42A1 scFv-mPE24. Finally, the antigen-binding patterns of 32A9 and 42A1 were compared under neutral and acidic pH conditions.

**Results:**

Although 32A9 and 42A1 had similar antigen binding capacities and internalization rates, 32A9 scFv-mPE24 had superior antitumor activity compared to 42A1 scFv-mPE24. We found that 32A9 scFv-mPE24 exhibited faster degradation and drove efficient free toxin release compared to 42A1 scFv-mPE24. These phenomena were determined by the different degradation behaviors of 32A9 scFv-mPE24 and 42A1 scFv-mPE24 in lysosomes. Moreover, 32A9 was sensitive to the low-pH environment, which made the 32A9 conjugate easily lose antigen binding and undergo degradation in lysosomes, and the free toxin was then efficiently produced to exert cytotoxicity, whereas 42A1 was resistant to the acidic environment, which kept the 42A1 conjugate relatively stable in lysosomes and delayed the release of free toxin.

**Conclusions:**

These results showed that a low pH-sensitive antibody-based immunotoxin degraded faster in lysosomes, caused effective free toxin release, and led to improved cytotoxicity compared to an immunotoxin based on a normal antibody. Our findings suggested that a low pH-sensitive antibody might have an advantage in the design of immunotoxins and other lysosomal degradation-dependent antibody conjugate drugs.

## Introduction

Immunotoxins are chimeric molecules that consist of antibody fragments fused to toxin fragments. Immunotoxins recognize the surface antigen of tumor cells and are internalized with the antigen into tumor cells. Internalized immunotoxins are degraded in lysosomes, and the free toxin fragment is released into the cytosol [1]. The released free toxin fragment of *Pseudomonas* exotoxin (PE)-type immunotoxin catalyzes the ADP-ribosylation of elongation factor 2, inhibits protein synthesis, and leads to apoptosis [2]. In general, immunotoxins exhibit effective cytotoxicity against tumor cells, which makes them an attractive strategy for cancer therapy [3–6]. Currently, an immunotoxin against CD22 has achieved exciting success as a treatment for hairy cell leukemia and was approved by the FDA [7], and several immunotoxins targeting solid tumors are also undergoing clinical evaluation [8–11].

The cytotoxicity of immunotoxins is directly mediated by the free toxin fragment released from lysosomes. However, most immunotoxins are entirely degraded (~ 95%) by a variety of hydrolytic enzymes in acidic lysosomes, and only a small portion of immunotoxins are digested into free toxin that escapes into the cytosol [12]. The details of immunotoxin degradation and free toxin escape into the cytosol remain unclear. Therefore, overcoming the low intracellular utilization of immunotoxins is of great interest in improving the antitumor activity of immunotoxins. Many studies have focused on the engineering of toxin fragments to attenuate immunogenicity [13, 14], reduce off-target toxic effects [15], and extend the half-life of immunotoxins [16, 17]. To date, the optimized PE toxin fragment only maintains the furin cleavage site and domain III, which are important for cytotoxic activity and directly mediate the catalytic reaction of immunotoxins, respectively [18, 19]. Those optimized sequences of the PE fragment are unlikely to be further engineered to improve the intracellular use of immunotoxins without compromising their cytotoxic activity. However, whether antibody fragments can regulate the cytotoxicity of immunotoxins has not been well explored.

In fact, many studies have attempted to develop antibodies with different characteristics to optimize the therapeutic effects of antibody drugs, such as antibodies against different epitopes of HER2, which were reported to internalize with different efficiencies [20]. Additionally, modifying the affinity of antibodies in an acidic environment affected the behavior of the antibody drug in lysosomes. For example, by reducing the affinity of pertuzumab at acidic pH, its related antibody‒drug conjugates (ADCs) showed increased lysosomal delivery and cytotoxicity toward tumor cells [21]. These findings all showed the influence of antibodies on the behavior and functions of antibody conjugates.

Our previous studies established a series of immunotoxins targeting glypican-3 (GPC3) [3], a cell surface antigen that is specifically expressed in hepatocellular carcinoma (HCC) [22]. We selected an antibody targeting the Wnt-binding site of GPC3 to construct a novel anti-GPC3 immunotoxin, which exhibited effective antitumor activity via PE toxin-mediated cytotoxicity and antibody fragment-mediated blocking of cancer signaling [3, 23]. Moreover, we replaced PE38 with mPE24 to deduce the undesirable immunogenicity of the PE toxin and successfully achieved improved antitumor activity by increasing the in vivo safety of immunotoxin [24].

In the current study, we constructed immunotoxins using two anti-GPC3 antibodies with similar affinity and endocytosis efficiency and found that they exhibited significantly different in vitro and in vivo antitumor activities. These two immunotoxins exhibit different lysosomal behaviors, which results in differences in their intracellular protein stabilities and free toxin release. Further investigation indicated that the antigen-binding capacity of these two antibodies varied at acidic pH, which may be the main factor to affect immunotoxin protein stability in the lysosome and the antitumor activity of the immunotoxin.

## Methods and materials

### Clinical samples

Normal liver tissue and HCC tumor tissue specimens were obtained from 2 male patients who underwent surgical resection for HCC at Jiangsu Taizhou People’s Hospital (Taizhou, Jiangsu) between November 2022 and March 2023. The histologic grade of tumor differentiation was at II, according to the Edmondson grading system. None of the patients had multiple tumor, satellite nodule, vascular invasion or distant metastasis. The patients did not receive anticancer therapies before the surgical operation.

### Cell lines

Huh-7 cells were a kind gift from Dr. Xin-Wei Wang at the National Cancer Institute (Bethesda, MD, USA). A431, Hep3B and HEK293T cells were purchased from American Type Culture Collection (Manassas, VA, USA). All cells were cultured in DMEM (HyClone, Logan, UT, USA) supplemented with 10% fetal bovine serum (VACCA, St. Louis, MO, USA), 100 U/ml penicillin, and 0.1 mg/ml streptomycin (HyClone, Logan, UT, USA) and were incubated in 5% CO_2_ at 37 °C. A431 cells were engineered to highly express GPC3 via transfection with a plasmid encoding full-length GPC3. All cell lines were evaluated and validated by their morphology and growth rate. All cell lines were confirmed to be free of mycoplasma contamination using PCR with specific primers.

### Plasmids, proteins, and antibodies

The sequence of full-length GPC3 was cloned into the pLVX vector. For protein purification, truncated GPC3 (Q25-S550) was fused to a human Fc tag and cloned into the pFUSE vector. The 32A9 or 42A1 scFv sequence, isolated from the Tomlinson I library to bind human GPC3 [25, 26], was fused to the human Fc tag and cloned into the pFUSE vector. The 32A9 or 42A1 heavy chain variable region and light chain variable region sequences were amplified by adding the IL-2 signal peptide and were inserted into the expression vectors pFUSE-CHIg-HG1 and pFUSE2-CLIg-hk (Invitrogen, San Diego, CA), respectively. All pFUSE plasmids were identified by sequencing and then transfected into 293 T cells. After collecting the supernatant, protein purification was performed using a protein A affinity column (GE Healthcare, Milwaukee, WI, USA). GPC3-His and GPC5-His proteins were purchased from R&D (Minneapolis, MN, USA).

YP7, which is a control mouse monoclonal antibody against GPC3 [27], was used to evaluate GPC3 expression by immunohistochemistry staining, flow cytometry and ELISA.

### Structural modeling

The 32A9 or 42A1 scFv model and scFv-mPE24 model was submitted to the protein structure modeling web server Phyre2 [28]. PyMOL was used to analyze and render structural models [29].

### ELISA

GPC3-hFc or GPC3-His protein (5 μg/ml) was used to coat ELISA wells at 4 °C overnight. The wells were blocked with PBS buffer containing 3% milk and 0.05% Tween-20 for 0.5 h at 37 °C. The antibodies or phages were added to the wells and incubated at 37 °C for 0.5 h. After washing with PBS (containing 0.05% Tween-20) buffer for 3 times, goat anti-human Fc, kappa chain HRP antibody (for 32A9 or 42A1 IgG) (Life Tech, Peoria, IL, USA) or rabbit anti-M13 HRP antibody (for phage) (GE Healthcare, Milwaukee, WI, USA) was added to the wells and incubated at 37 °C for 0.5 h. TMB and sulfuric acid were added to detect the OD_450nm_ value.

### Western blotting

The cells were lysed with RIPA/PMSF/protease inhibitor buffer on ice and centrifuged to extract the protein. For the non-reduced analysis, the native protein is treated with SDS prior to separation to mask the protein native charges. For reduced analysis, the sample is treated with SDS and dithiothreitol (DDT) to reduce the native protein structure. Then the total proteins were separated on SDS-PAGE and transferred onto the PVDF membrane (Merck Millipore, Tullagreen, Carrigtwohill, Ireland). After blocking with 5% milk overnight, the PVDF membranes were incubated with primary antibodies for 1 h at room temperature. Anti-β-actin HRP (Sigma, St. Louis, MO, USA) was used as a loading control. The ImageJ software was used to quantify the gray value of the protein bands.

### Flow cytometry

Cells were trypsinized into single cell suspensions and being fully dispersed by a pipette after centrifugation and were counted using a cell counting plate. 5 × 10^5^ A431-GPC3 cells were incubated with 5 µg/ml of the indicated antibodies or different concentrations of immunotoxins for 1 h on ice and then incubated with a 1:200 dilution of anti-human PE antibody (Thermo, Pudong New Area, Shanghai) or rabbit anti-Pseudomonas exotoxin A antibody (Sigma, St. Louis, MO, USA) and goat anti-rabbit PE (Abcam, Cambridge, England) for 1 h on ice. After washing with PBS buffer, unstained cells and labeled cells with second fluorescence antibody only were defined as negative cell group, then the positive cell group was gated and continuously collected to 1 × 10^4^ cells using FACS Calibur (BD Biosciences, San Jose, CA, USA) for further analysis by using FlowJo 7.6.

### Antibody internalization detection

A total of 3 × 10^5^ A431-GPC3 cells were seeded into 24-well plates and cultured overnight at 80–90% density. A431-GPC3 cells were coincubated with 50 µg/ml Alexa 488-labeled 32A9 IgG or Alexa 488-labeled 42A1 IgG immediately (0), 0.5, 1, 2, and 4 h at 37 °C. A431-GPC3 cells were stripped with 0.2 M glycine buffer (pH 2.5) after washing with PBS buffer. The digested cell suspensions were analyzed using FACS Calibur (BD Biosciences, San Jose, CA, USA).

### Immunotoxin purification

The sequence of 32A9 or 42A1 scFv was fused to a truncated and deimmunized *Pseudomonas* exotoxin (mPE24) fragment and then cloned into the pMH212 vector [13]. 100 uL of BL21 competent cells (Weidi Biotechnology, Minhang District, Shanghai) were transfected with the plasmid and induced with 1 mM IPTG for 1.5 h at 37 °C. The inclusion body was collected, washed, and lysed using lysozymes to obtain the raw extraction of the recombinant protein. The recombinant protein was denatured and refolded, and the buffer was exchanged via overnight dialysis. The refolded recombinant protein was acquired using chromatography with ÄKTA pure (GE Healthcare, Milwaukee, WI, USA): we used Q-Sepharose and Mono-Q (GE Healthcare, Milwaukee, WI, USA) ion-exchange chromatography for separating molecules on the basis of charge, and finally eluted immunotoxin protein into PBS buffer using a TSK (Tosoh, Changning, Shanghai) gel-filtration step that separated molecules on the basis of size, and determined protein concentration, aliquot, and freeze at 70 °C.

### Co-localization analysis

A total of 5 × 10^4^ A431-GPC3 cells were seeded into confocal dish and cultured overnight at 70–80% density. After incubating A431-GPC3 cells with 50 µg/ml immunotoxin on ice for 1 h, the cells were washed with PBS and labeled with anti-Pseudomonas exotoxin A antibody (Sigma, St. Louis, MO, USA) and Alexa647-conjugated goat anti-rabbit (Invitrogen, San Diego, CA, USA) on ice for 1 h. The treated cells were coincubated with DMEM immediately (0), 1 and 3 h at 37 °C to internalize labeled immunotoxins. Thereafter, the cells were incubated with lysosome tracer (Beyotime, Songjiang, Shanghai) for 1 min and DAPI (Beyotime, Songjiang, Shanghai) in the dark for 1 min and observed under laser confocal microscope in randomly selected fields.

### Antibody and immunotoxin stability detection

A total of 3 × 10^5^ A431-GPC3 cells were seeded into 24-well plates and cultured overnight at 80–90% density. After incubating A431-GPC3 cells with 50 μg/ml immunotoxin at 37 °C for 1 h, the cells were washed with PBS and 0.2 M glycine buffer (pH 2.5). The treated cells were coincubated with DMEM immediately (0), 0.5, 1, 2, and 4 h at 37 °C to degrade accumulated immunotoxin. A431-GPC3 cells were washed and lysed for Western blot analysis. The antibodies used included anti-*Pseudomonas* exotoxin A antibody (Sigma, St. Louis, MO, USA), anti-rabbit HRP (Jackson Laboratory, Bar Harbor, ME, USA) and anti-β-actin HRP (Sigma, St. Louis, MO, USA). A431 cells were also treated as negative control.

To block lysosomal degradation, 50 µM chloroquine (MCE, St. Monmouth Junction, NJ, USA) was added to the wells for 12 h before incubating the cells with immunotoxin during immunotoxin degradation.

### Free toxin release detection

A total of 3 × 10^5^ A431-GPC3 cells were seeded into 24-well plates and cultured overnight at 80–90% density. A431-GPC3 cells were coincubated with 50 µg/ml immunotoxin immediately (0), 0.5, 1, 2, 4, and 8 h at 37 °C to accumulate free toxin. A431-GPC3 cells were stripped with 0.2 M glycine buffer (pH 2.5) and lysed for Western blot analysis. The antibodies used included anti-*Pseudomonas* exotoxin A antibody (Sigma, St. Louis, MO, USA), anti-rabbit HRP (Jackson Laboratory, Bar Harbor, ME, USA) and anti-β-actin HRP (Sigma, St. Louis, MO, USA). A431 cells were also treated as a negative control.

To block lysosomal degradation, 50 µM chloroquine (MCE, St. Monmouth Junction, NJ, USA) was added to the wells for 12 h before incubating cells with immunotoxin during immunotoxin degradation.

### Cytotoxicity of immunotoxins

A total of 1 × 10^4^ cells were seeded into 96-well plates and cultured overnight at 80–90% density. Different concentrations of immunotoxin were added to the wells. After 72 h, CCK8 (Beyotime, Songjiang, Shanghai) is added to each well, and the incubation is carried out for 1 h at 37 °C. The absorbance of the sample at 450 nm is measured. Cytotoxicity is expressed as 50% inhibition of cell viability, which is halfway between the level of viability in the absence of toxin and that in the presence of 10 mg/ml of cycloheximide.

### Animal tests

All animal experiments were approved by the Institutional Animal Care and Use Committee (IACUC) of Nanjing Medical University under the ethical review number “No. IACUC-2107004”. 60 four- to six-week-old female BALB/c nude mices (BALB/cJGpt-Foxn1nu/Gpt) were purchased from GemPharmatech (Guangzhou, China). To evaluate the antitumor activity of the immunotoxins, 5 × 10^6^ A431-GPC3 cells were subcutaneously inoculated into five-week-old nude mice. When the average tumor size reached ~ 100 mm^3^, according to the order of tumor size, mice bear moderate tumor were selected for immunotoxin treatment, n = 5/group. The mice were treated daily with 2.5 or 5 mg/kg immunotoxin via i.v. injection. Tumor dimensions were determined using calipers, and tumor volume was calculated using the formula *V* = 1/2 *ab*^2^, where a and b represent tumor length and width, respectively.

### Statistical analysis

Representative results were obtained from at least three independent experiments. All group data (except otherwise indicated) are expressed as the means ± standard deviations (SD) of a representative experiment performed at least in triplicate, and similar results were obtained in at least 3 independent experiments. All statistical analyses were performed using GraphPad Prism 5.0. Two-tailed Student’s t tests and paired Student’s t test were used for statistical analyses, and *P < 0.05 indicated significance.

## Results

### Immunotoxin against GPC3 effectively induced cytotoxicity in HCC cells

**Fig. 1:**
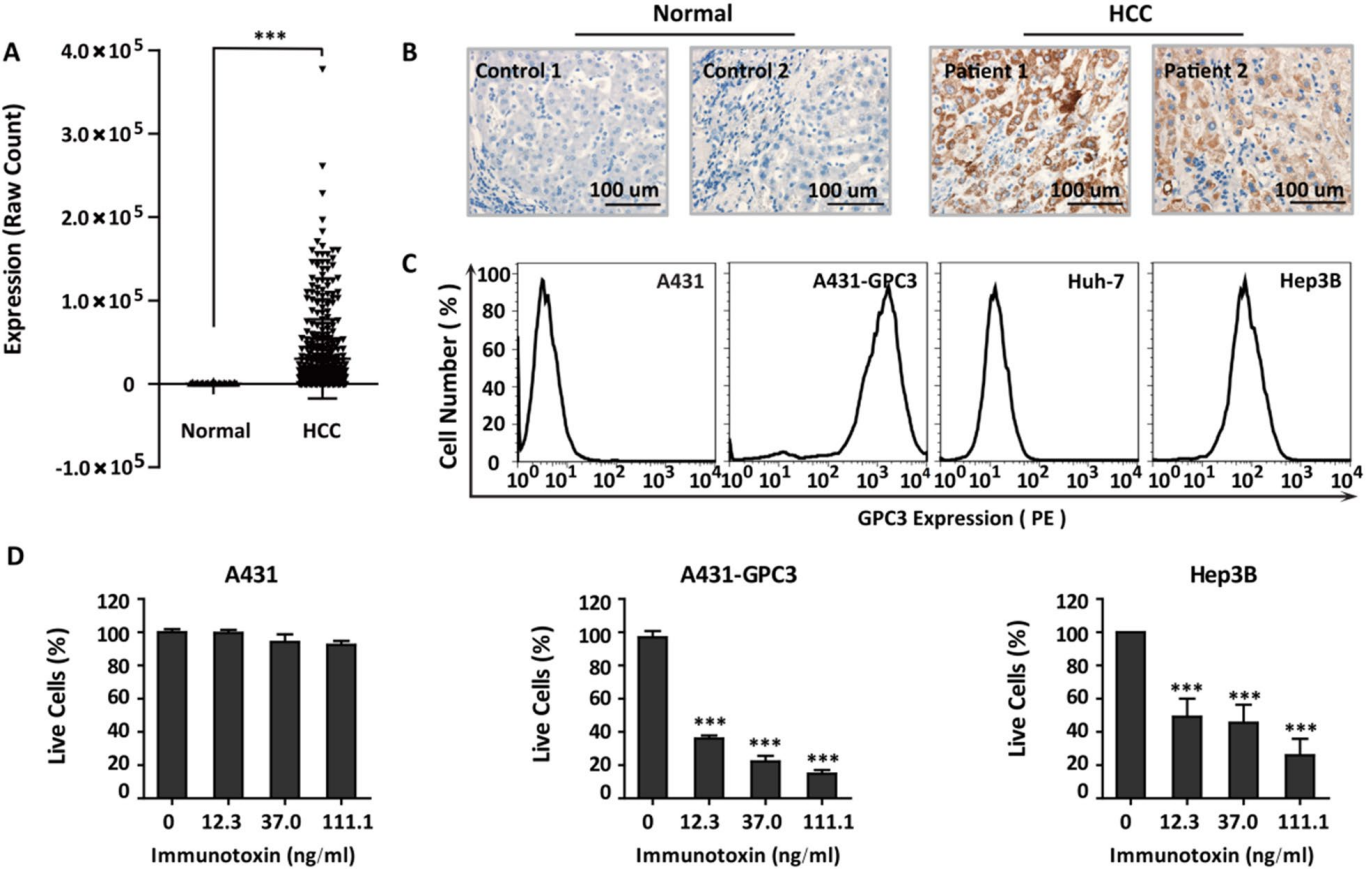
GPC3 is a potent target for developing immunotoxin strategies in liver cancer. **A** The transcriptional expression of GPC3 in liver cancer from The Cancer Genome Atlas Program (TCGA database). n = 369 in the LIHC (liver hepatocellular carcinoma) group, n = 50 in the normal liver tissue group, Values represent the mean ± SD, ***P < 0.0001 (two-tailed Student’s *t*-test). **B** Immunohistochemical staining of GPC3 in HCC patient tumor tissues and normal liver tissues. **C** Flow cytometry was used to detect the cell surface expression of GPC3 in HCC cells. A431 cells and A431-GPC3 cells are shown as negative and positive controls, respectively. **D** WST assay to detect the cytotoxicity of the anti-GPC3 immunotoxin (HN3-mPE24). Values represent the mean ± SD, n = 3 individual tests, with ***P < 0.001 (two-tailed Student’s *t*-test)

HCC is the major subtype of primary liver cancer, but it lacks effective treatment. GPC3 is an oncofetal antigen that is specifically expressed in HCC patients [22, 30]. Analysis of the TCGA database and clinical samples of HCC patients revealed that GPC3 was abnormally expressed in HCC patients but not in normal liver tissue (Table 1; Fig. 1A, B). GPC3 exhibited strong membrane expression in HCC cells, which makes it a potent target for immunotherapy (Fig. 1C). Due to its rapid endocytosis, GPC3 is also a suitable target for antibody conjugates [3, 31]. HN3-mPE24, which is the immunotoxin against GPC3 developed in our previous study [24], effectively killed GPC3-positive HCC cells (Fig. 1D). Therefore, we took advantage of the anti-GPC3 immunotoxin to investigate the regulatory effect of the antibody on the function of the immunotoxin.

**Table 1:** Characteristics of HCC patients

Case	Gender	Age	Edmondson Grade	Tumor size (cm)	Tumor multiplicity	Vascular invasion	Satellite nodule	TNM stage	Metastasis
#1	Male	60	II	2.8	1	Absent	0	1	Absent
#2	Male	50	II	8	1	Absent	0	2	Absent

TNM: tumor-node-metastasis

### 32A9 scFv-mPE24 has more effective antitumor activity than 42A1 scFv-mPE24 in vitro and in vivo

**Fig. 2:**
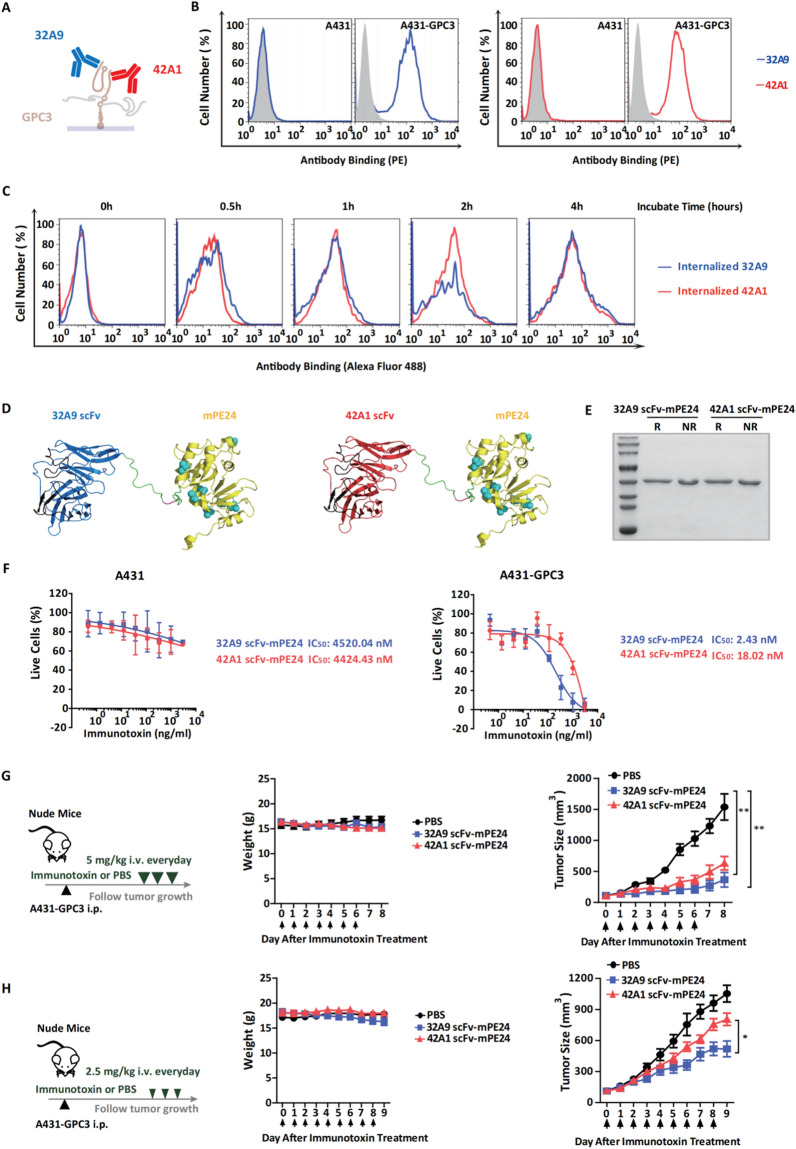
32A9 scFv-mPE24 and 42A1 scFv-mPE24 showed different antitumor activities in vitro and in vivo. **A** Schematic diagram of 32A9 (blue), 42A1 (red) antibody and GPC3 molecule (brown). **B** Flow cytometry to detect the antibody-binding activity and specificity to A431-GPC3 cells. Gray: Negative control, blue: 32A9, red: 42A1. **C** Flow cytometry to detect internalized Alexa 488-labeled 32A9 (blue) and 42A1 (red). **D** Schematic diagram of 32A9 scFv-mPE24 and 42A1 scFv-mPE24. PE: *Pseudomonas exotoxin* A. **E** SDS‒PAGE to show the purified scFv-mPE24. R: reducing. NR: nonreducing. **F** WST assay to detect the cytotoxicity of 32A9 scFv-mPE24 and 42A1 scFv-mPE24. The dashed line indicates the IC_50_ value. Values represent the mean ± SD, n = 3 individual tests. Nude mice were subcutaneously injected with five million A431-GPC3 cells and then treated with 5 mg/kg (**G**) or 2.5 mg/kg (**H**) immunotoxin every day. Arrow indicated individual injections. Body weight of the mice was recorded, n = 5/group, Values represent mean ± SEM, with *P < 0.05, **P < 0.01 (paired Student’s t-test). Tumor growth after immunotoxin treatment was monitored by measuring tumor size. n = 5/group. Values represent the mean ± SEM, with *P < 0.05, **P < 0.01 (paired Student’s *t*-test)

We isolated two human antibodies that target GPC3, 32A9 and 42A1, by phage display in our previous work [25, 26] (Fig. 2A). The two antibodies showed similar binding activity for cell surface GPC3 (Fig. 2B) and triggered similar endocytosis when they targeted the antigen (Fig. 2C). These results indicated that both 32A9 and 42A1 can be used to construct anti-GPC3 antibody conjugates. We fused the single-chain fragment of the variable region of 32A9 and 42A1 to mPE24, a deimmunized PE fragment [13], to construct anti-GPC3 immunotoxins (Fig. 2D, E). To validate the functions of 32A9 scFv-mPE24 and 42A1 scFv-mPE24, we performed a WST assay to examine their in vitro cytotoxicity in GPC3-positive cells. Both 32A9 scFv-mPE24 and 42A1 scFv-mPE24 exhibited specific cytotoxicity on A431-GPC3 cells but not A431 cells. Moreover, 32A9 scFv-mPE24 showed more potent cytotoxicity than 42A1 scFv-mPE24 (Fig. 2F). We performed in vivo animal studies to further evaluate the antitumor effect of 32A9 scFv-mPE24 and 42A1 scFv-mPE24 immunotoxins. Nude mice were inoculated with A431-GPC3 cells subcutaneously and treated daily with different doses of immunotoxin. When mice were treated with 5 mg/kg immunotoxins, both the 32A9 scFv-mPE24-treated group and 42A1 scFv-mPE24-treated group showed significant tumor elimination compared to the PBS-treated group (Fig. 2G). When we reduced the dose of immunotoxin to 2.5 mg/kg, 32A9 scFv-mPE24-treated mice exhibited more effective antitumor activity than 42A1 scFv-mPE24-treated mice (Fig. 2H), which was consistent with our in vitro observations. The mice treated with 5 mg/kg (Fig. 2G) or 2.5 mg/kg (Fig. 2H) of immunotoxin showed only moderate body weight loss and all the mice survived after the immunotoxin administration was completed.

### The intracellular stability and release of free toxin differ between 32A9 scFv-mPE24 and 42A1 scFv-mPE24

**Fig. 3:**
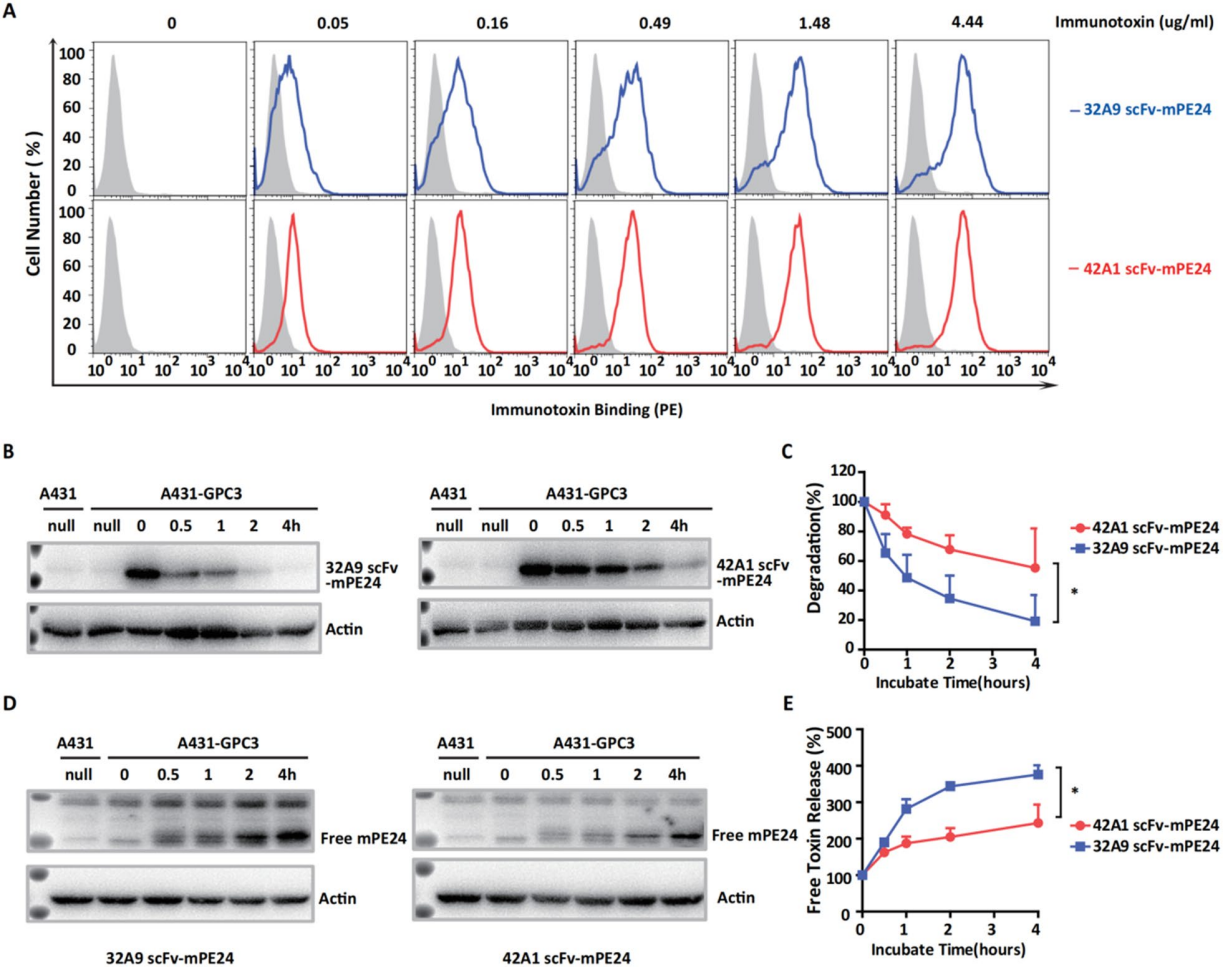
32A9 scFv-mPE24 and 42A1 scFv-mPE24 showed different protein stabilities and related free toxin release. **A** Binding activity of immunotoxin to A431-GPC3 cells. Gray: secondary antibody only as a negative control. **B** Western blotting was used to detect the stability of the internalized immunotoxins. (**C**). Statistical analysis of (**B**). Values represent the mean ± SD, n = 3 individual tests, with *P < 0.05 (paired Student’s *t*-test). **D** Western blot to detect the accumulation of free toxin after immunotoxin internalization. **E** Statistical analysis of (**D**). Values represent the mean ± SD, n = 3 individual tests, with *P < 0.05 (paired Student’s *t*-test)

Since 32A9 and 42A1 showed similar binding capacity and endocytosis efficiency on cells, we suspected that the different antitumor activities of 32A9 scFv-mPE24 and 42A1 scFv-mPE24 might be caused by their intracellular behavior. Specifically, 32A9 scFv-mPE24 and 42A1 scFv-mPE24 maintained similar binding capabilities on GPC3-positive cells, suggesting that the different cytotoxicities of these two immunotoxins were not triggered by the antigen-binding step (Fig. 3A). Therefore, we directly detected the intracellular stability of these two conjugates and found that 32A9 scFv-mPE24 was immediately degraded after internalization, but 42A1 scFv-mPE24 showed better intracellular stability (Fig. 3B, C). Because the free toxin fragment directly inhibited protein synthesis by catalyzing the ADP-ribosylation of elongation factor 2, we next examined whether 32A9 scFv-mPE24 had faster free toxin accumulation following its faster degradation. As expected, free toxin accumulated more efficiently in the cells treated with 32A9 scFv-mPE24 (Fig. 3D, E). These observations all indicated that the superior antitumor activity of 32A9 scFv-mPE24 might be induced by its faster degradation, which triggered the quick accumulation of free toxin in cells.

### The degradation difference between 32A9 scFv-mPE24 and 42A1 scFv-mPE24 occurred in lysosomes

**Fig. 4:**
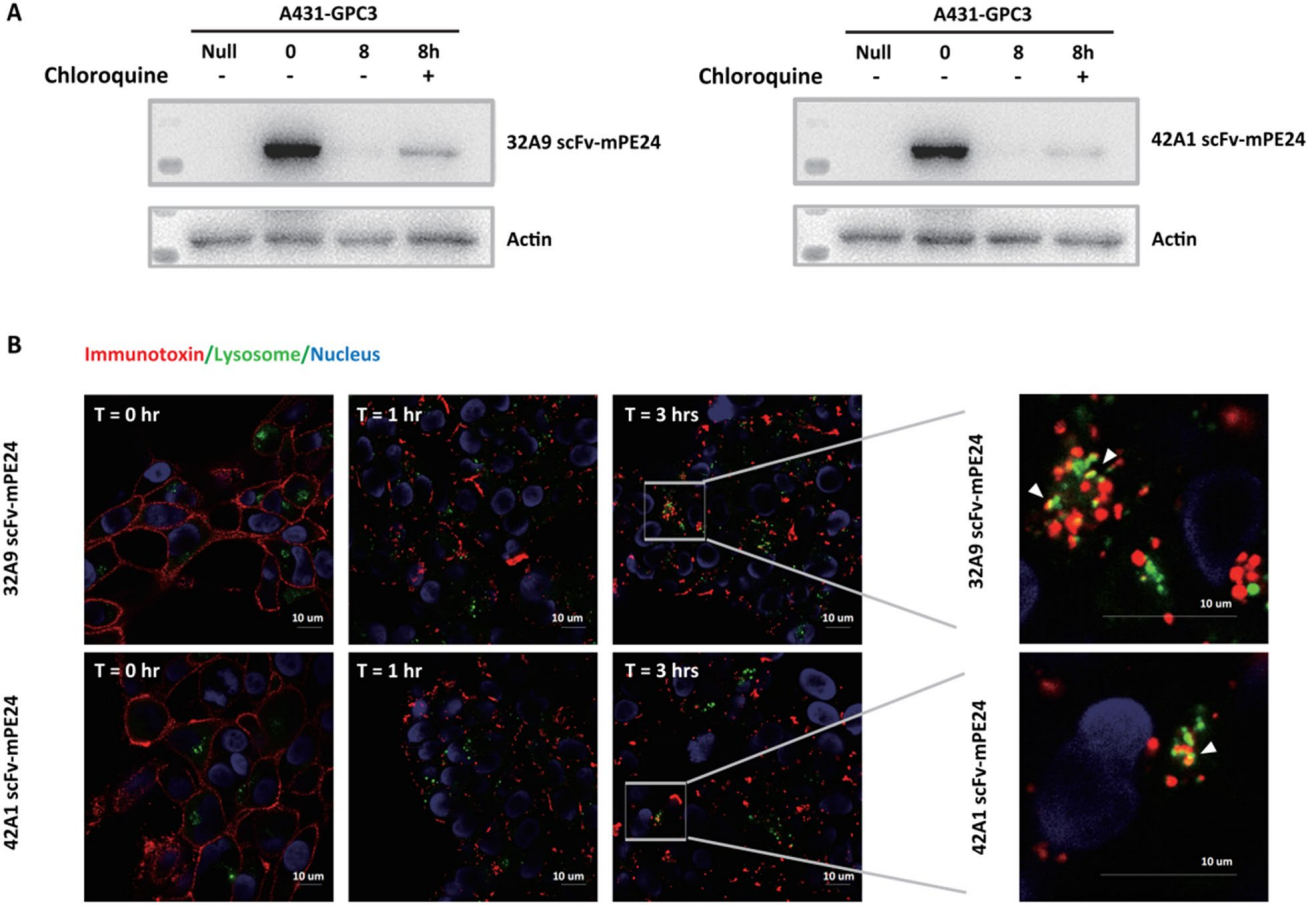
32A9 scFv-mPE24 and 42A1 scFv-mPE24 entered into lysosome by similar intracellular trafficking. **A** Western blot to detect the stability of internalized immunotoxins in A431-GPC3 cells with or without lysosomal inhibitor treatment. **B** Time-dependent internalization and trafficking of 32A9 and 42A1 scFv-mPE24 in A431-GPC3 cells. 32A9 or 42A1 scFv-mPE24 visualized using anti-PE toxin antibody and Alexa647 labeled secondary antibody (red). Nuclei and lysosomes were stained with DAPI (blue) and lysosome tracer (green), respectively. Arrow indicated co-location of immunotoxin and lysosome

**Fig. 5:**
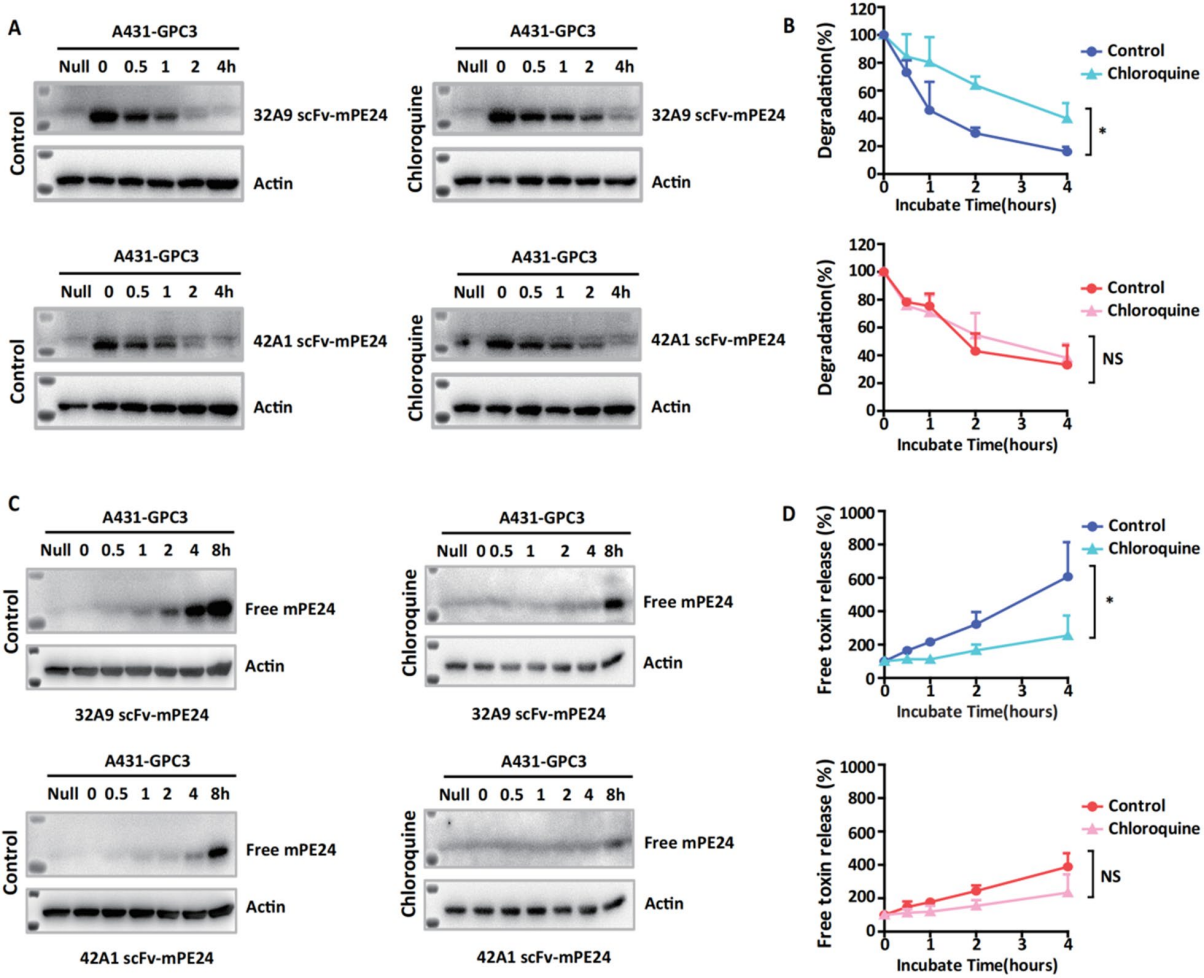
The lysosome degradation and related free toxin release of 32A9 scFv-mPE24 were sensitively affected by chloroquine treatment. **A** Western blot to detect the stability of internalized immunotoxins in A431-GPC3 cells with or without lysosomal inhibitor treatment. **B** Statistical analysis of (**A**). Blue: 32A9 scFv-mE24, red: 42A1 scFv-mPE24. Values represent the mean ± SD, n = 3 individual tests, with *P < 0.05 (paired Student’s *t*-test). **C** Western blot to detect free toxin accumulation after immunotoxin internalization into A431-GPC3 cells treated with or without lysosomal inhibitors. **D** Statistical analysis of (**C**). Blue: 32A9 scFv-mE24, red: 42A1 scFv-mPE24. Values represent the mean ± SD, n = 3 individual tests, with *P < 0.05 (paired Student’s *t*-test)

Lysosomes are the main degradative compartments of immunotoxins. When we treated cells with chloroquine, the degradation of 32A9 scFv-mPE24 and 42A1 scFv-mPE24 was rescued (Fig. 4A), and immunofluorescence staining of the uptake of immunotoxins under confocal microscopy confirmed the localization of 32A9 scFv-mPE24 and 42A1 scFv-mPE24 in the lysosomes from cell membrane after internalization (Fig. 4B). These observations indicated that 32A9 scFv-mPE24 and 42A1 scFv-mPE24 was degraded in lysosomes. Therefore, we suspected that the different degradation behaviors of 32A9 scFv-mPE24 and 42A1 scFv-mPE24 were caused by their sensitivity to acidic lysosomal hydrolysis. We detected immunotoxin stability by treating A431-GPC3 cells with chloroquine, an inhibitor preventing lysosomal acidification [32]. Our results showed that the lysosome inhibitor dramatically blocked immunotoxin degradation in 32A9 scFv-mPE24-treated cells, but these inhibitors induced no obvious blocking effects in 42A1 scFv-mPE24-treated cells (Fig. 5A, B). Accordingly, the free-toxin accumulation of 32A9 scFv-mPE24-treated cells was significantly attenuated when cells were treated with chloroquine, whereas that of 42A1 scFv-mPE24-treated cells was not obviously affected (Fig. 5C, D). Since the only difference between these two immunotoxins is the antibody fragment, we suspected that the acidic responsiveness of 32A9 and 42A1 might be the major cause of the different degradation behaviors of the two immunotoxins.

### The antigen-binding affinity of 32A9 decreased dramatically under acidic conditions, whereas that of 42A1 remained stable

**Fig. 6:**
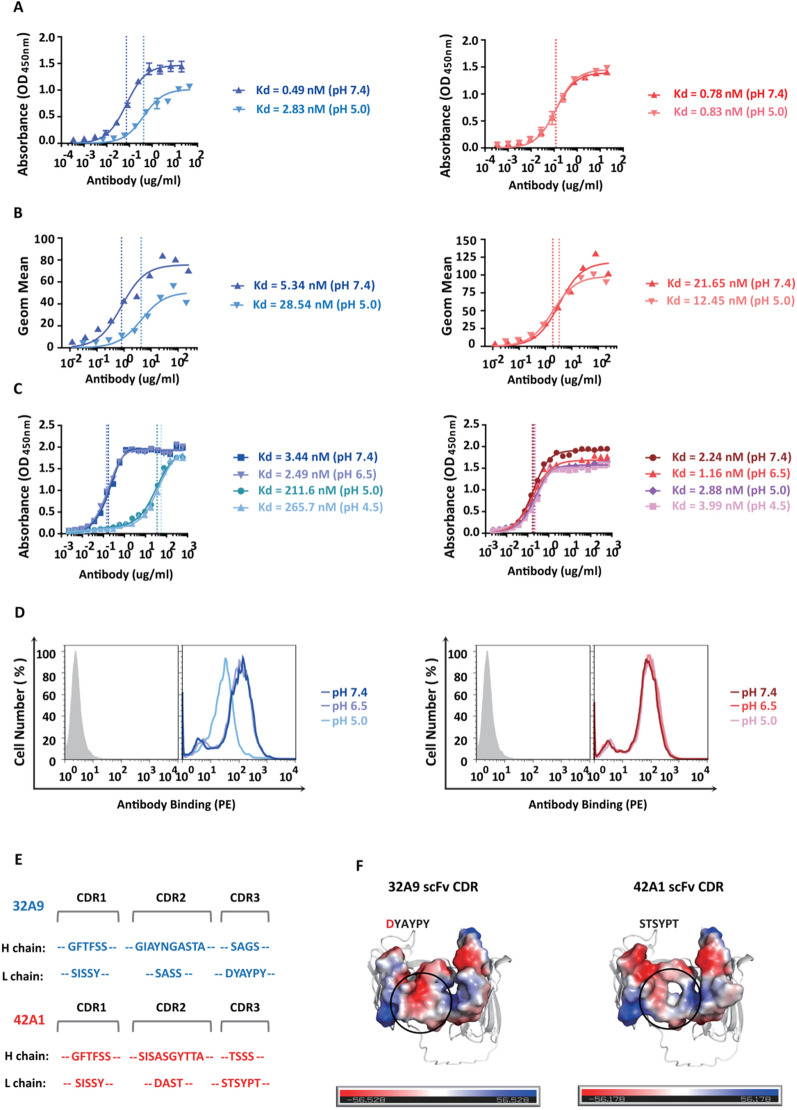
32A9 and 42A1 showed different antigen-binding capacities in acidic pH. Affinity analysis of 32A9 and 42A1 to purified GPC3 protein (**A**) and A431-GPC3 cells (**B**) under different pH environments, contains pH 7.4 and pH 5.0. Blue: 32A9, red: 42A1. Values represent the mean ± SD, n = 3 individual tests in (**A**). **C** Affinity analysis of 32A9 and 42A1 to purified GPC3 protein under different pH environments, contains pH 7.4, pH 6.5, pH 5.0 and pH 4.5. Blue: 32A9, red: 42A1. n = 1 individual test. **D** Binding activity of 32A9 and 42A1 to A431-GPC3 cells under different pH environments, contains pH 7.4, pH 6.5 and pH 5.0. Blue: 32A9, red: 42A1. Gray: secondary antibody only as a negative control. **E** Alignment of 32A9 and 42A1 CDR sequence. **F** Surface charge distribution of scFv antibodies. The structure model of 32A9 and 42A1 scFv generated by Phyre2. Surface charge is calculated and visualized by PyMOL. Blue: Positive charge, Red: Negative charge

To validate our hypothesis, we measured the affinity of two antibodies under acidic conditions near the lysosome and in a neutral environment and found that the binding activity of the purified 32A9 antibody to GPC3 protein and GPC3-positive cells dramatically decreased under acidic conditions compared to its activity under neutral conditions (approximately 5–6-fold). In contrast, the binding activity of the purified 42A1 antibody did not show much difference (Fig. 6A, B). The results suggested that the different binding capacities of 32A9 and 42A1 in acidic pH caused the difference in the degradation behavior of the two immunotoxins. To exam whether the acidic tumor microenvironment would affect the antibody recognition or not, we detected the antigen binding activities of 32A9 and 42A1 under pH 7.4, pH 6.5 and pH 5.0 to mimic normal tissue, tumor micro environment [33], and matured lysosome [34], respectively. The results showed that the binding activity of two antibodies did not change under pH 6.5, and only when the pH reduced to 5.0, the binding activity of 32A9 dramatically reduced but not to 42A1(Fig. 6C, D). All these results supported that 32A9 worked as a pH dependent antibody when it internalized into lysosome, and its antigen recognition would not be affected in tumor microenvironment. In theory, the interactions that pH changes affect contains salt bridges and hydrogen bonding in protein folding and protein–protein interaction [35], we carefully compared the CDR sequences of two antibodies. We found that the light chain CDR3 of 32A9 has an aspartate where 42A1 has uncharged residues, suggesting this region might contribute to the unique pH response of 42A1(Fig. 6E, F).

**Fig. 7:**
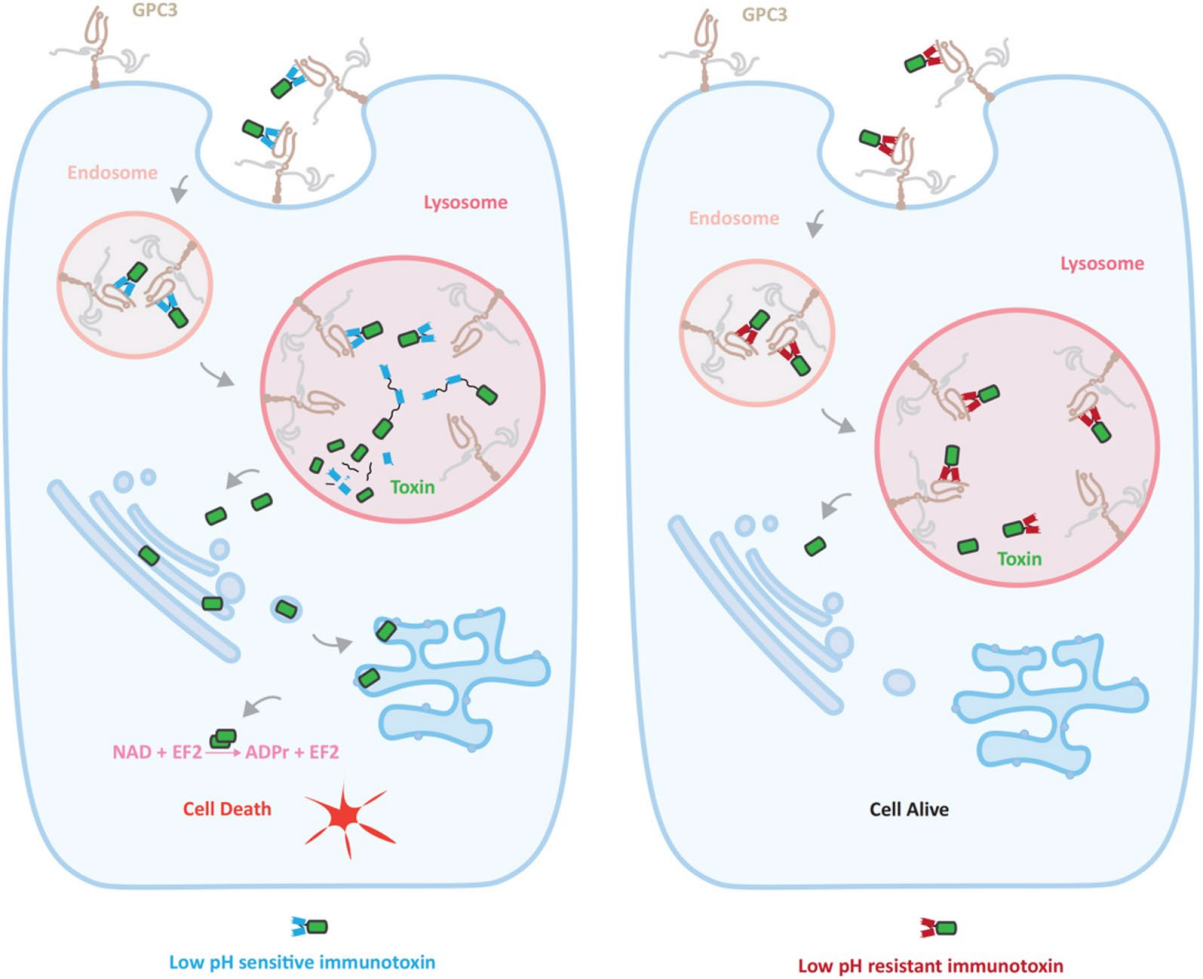
Working model of the regulatory effect of low pH-responsive antibodies on the intracellular behavior of immunotoxins. Low pH-sensitive antibody-based immunotoxins lead to fast degradation in lysosomes, which accelerates the accumulation of free toxin and produces effective cytotoxicity in tumor cells

Taken together, our results showed that the acidic responsiveness of the antibody might determine the lysosome degradation behavior of the immunotoxin, which could consequently affect its cytotoxicity. When transported into lysosomes, 32A9 scFv-mPE24 underwent faster denaturation and degradation due to quick dissociation from the antigen, triggering the efficient accumulation of free toxin and inducing effective cytotoxicity. However, 42A1 scFv-mPE24 remained stable in lysosomes and did not release free toxin efficiently because 42A1 still maintained good recognition of antigens in lysosomes (Fig. 7). Our study revealed the regulatory role of antibody fragments on the function of immunotoxins, which also provides a new concept in the design of immunotoxins and other lysosomal degradation-dependent antibody conjugate drugs.

## Discussion

We established pH-responsive immunotoxins to demonstrate the regulatory effect of antibody fragments on improving the intracellular utility of immunotoxins. Our results indicated that the pH-sensitive antibody-based immunotoxins exhibited a high efficiency of free-toxin release and led to better antitumor activity in vitro and in vivo.

PE, ricin, gelonin, and diphtheria are representative toxins used for constructing immunotoxins [2], but these immunotoxins exert cytotoxicity via different mechanisms; for example, PE toxin inhibits protein synthesis and cell death by catalyzing the irreversible ADP-ribosylation of eEF2, while ricin and gelonin inhibit protein synthesis by damaging ribosomes [36, 37]. However, most types of immunotoxins undergo Golgi-ER reverse transportation and are trafficked into endosomes/lysosomes for degradation. The free toxins are eventually transported to the cytosol [38]. The released free toxin ultimately exerts cytotoxicity in the cytosol; therefore, the efficiency of generating free toxin is the rate-limiting step of the efficient antitumor activity of immunotoxins. Based on these considerations, the immunotoxin with low pH-sensitive binding for antigen, which triggered the fast release of free toxin, unsurprisingly showed more potent antitumor effects in vitro and in vivo. However, the intracellular proteolysis process to generate free toxin varies between different types of immunotoxins, and whether the antibody with low pH-sensitive binding for antigen would also improve cytotoxicity when used to construct other types of immunotoxins will be examined in our future study.

Due to the poor clinical feedback and undesired toxicities, the anti-CD22 immunotoxin Lumoxiti will be permanently withdraw. However, the anti-GPC3 immunotoxin developed in the current study might exhibit superior advantages. Firstly, here we selected PE24 instead of PE38 to construct the antibody-toxin conjugate. The PE24 format immunotoxin dramatically reduced the vascular leak syndrome by removing the domain II of PE which mediated the unspecific binding to endothelium cells [39], and the improved adverse reactions of PE24-format immunotoxin would make patients to overcome the dose-limiting toxicity to accept higher dose of treatment. Moreover, GPC3 exhibited a quite efficient internalize behavior and could mediate a more efficient cytotoxicity [31].

Many studies have attempted to develop antibodies with pH-responsive binding to optimize their therapeutic effects [40, 41]. Similar to antibodies showing low pH sensitivity against IL-6R, PCSK9 and C5 were reported to dissociate from antigens in acidic endosomes [42–44], be captured by the neonatal Fc receptor (FcRn) and be recycled into the extracellular space, which improved the pharmacokinetics or pharmacodynamics of antibodies [45]. Moreover, pH-sensitive binding also enhanced the recycling rate of certain antigens [41]. A recent study found that ipilimumab, which is a clinically used antibody against CTLA-4 [46], markedly downregulated the cell surface expression level of CTLA-4 by disrupting CTLA-4 recycling, and the use of an antibody with pH-sensitive binding or increasing the pH sensitivity by introducing designed tyrosine-to-histidine mutations prevented antibody-triggered lysosomal CTLA-4 downregulation. Furthermore, this strategy also more effectively depleted tumor-infiltrating Tregs, which provided safer and more effective immunotherapy [47–50]. Many studies have developed antibodies with better binding affinity under acidic pH conditions than neutral pH conditions [40, 41]. These antibodies may induce fewer off-tumor effects and improve the safety of immunotherapy by taking advantage of the acidic tumor microenvironment. For example, an antibody with pH-responsive HER2 targeting had superior affinity and inhibited tumor spheroid growth more efficiently under acidic conditions [51]. Similarly, pH-responsive binding-based CAR-T cells function only within the acidic tumor microenvironment in a pH-dependent manner [52].

We isolated two distinct antibodies with similar antibody properties that showed different responsiveness to the target antigen under low pH conditions. We proved that the pH-responsive binding property of the antibody would primarily determine the intracellular stability and free toxin release of the immunotoxins. However, a more serious evaluation is needed to exclude whether the different intracellular behaviors of immunotoxins are epitope dependent, although two antibodies showed similar internalization rates after binding GPC3. Both 32A9 and 42A1 antibodies were isolated from the Tomlinson I phage library [53]. Due to the sequence characteristics of this library, the specific recognition of antigen was mainly determined by the heavy chain CDR2 and light chain CDR3 of antibody. In theory, the interactions effected by the change of pH contains salt bridges and hydrogen bonding in protein folding and protein–protein interaction [35]. Comparing the heavy chain CDR2 and light chain CDR3 of the two antibodies, we find that the light chain CDR3 of 32A9 has the sequence DYAYPY, whereas 42A1 has STSYPT. In this case, only 32A9 has an aspartate where 42A1 has uncharged residues. This difference might mediate the resistance of 42A1 to acidic pH when binding to GPC3. Based on these clues, we are considering to perform single-residue histidine scanning mutagenesis [54, 55] to obtain engineered 32A9 mutant variants with less pH sensitivity or 42A1 mutant variants with improved pH sensitivity for binding GPC3. The mutant variants, in parallel with their parental antibody, will be further evaluated in immunotoxin format because they all target the same epitope. Considering that the pH response differences of 32A9 and 42A1 were also possibly determined by the ionizable groups locating on the antigen epitope region, we would perform detailed analysis on each side and combined with histidine scanning mutagenesis in the future.

The liver is the major metabolic organ of the human body, and its detoxifying features generally cause resistance to chemo-drug treatment [56]. Therefore, immunotoxins may provide a feasible strategy for treating liver cancer because they cause cytotoxic killing via an enzymatic reaction to inhibit tumor cell protein synthesis as a recombinant protein drug instead of chemical drugs. Our findings provide new insight for designing effective immunotoxins and are meaningful for promoting the development of liver cancer therapy.

## Data Availability

The datasets used and/or analyzed during the current study are available from the corresponding author on reasonable request.

## Abbreviations

HCC: Hepatocellular carcinoma
LIHC: Liver hepatocellular carcinoma
GPC3: Glypican-3
scFv: single-chain fragment of the variable region
PE: Pseudomonas exotoxin A
ADCs: Antibody-drug conjugates
FcRn: Neonatal Fc receptor
